# A novel variant in *Salmonella* genomic island 1 of multidrug-resistant *Salmonella enterica* serovar Kentucky ST198

**DOI:** 10.1128/spectrum.03994-23

**Published:** 2024-04-30

**Authors:** Rattanaporn Intuy, Sirirak Supa-Amornkul, Bharkbhoom Jaemsai, Wuthiwat Ruangchai, Witthawat Wiriyarat, Soraya Chaturongakul, Prasit Palittapongarnpim

**Affiliations:** 1 Professor Pornchai Matangkasombut Center for Microbial Genomics (CENMIG), Department of Microbiology, Faculty of Science, Mahidol University, Bangkok, Thailand; 2 Department of Oral Microbiology, Faculty of Dentistry, Mahidol University, Bangkok, Thailand; 3 Department of Pre-Clinical and Applied Animal Science, Faculty of Veterinary Science, Mahidol University, Bangkok, Thailand; 4 Molecular Medical Biosciences Cluster, Institute of Molecular Biosciences, Mahidol University, Bangkok, Thailand; The University of Tennessee Knoxville, Knoxville, Tennessee, USA

**Keywords:** *Salmonella *genomic island 1 variant, *Salmonella *Kentucky ST198, chromosomal multiple-drug resistance, *bla_TEM-1b_*, *lnu*(F)

## Abstract

*Salmonella enterica* serovar Kentucky ST198 is a major health threat due to its resistance to ciprofloxacin and several other drugs, including third-generation cephalosporins. Many drug-resistant genes have been identified in the *Salmonella* genomic island 1 variant K (SGI1-K). In this study, we investigated the antimicrobial resistance (AMR) profile and genotypic relatedness of two isolates of ciprofloxacin-resistant (CIP^R^) *S*. Kentucky ST198 from poultry in Northeastern Thailand. We successfully assembled the complete genomes of both isolates, namely SSSE-01 and SSSE-03, using hybrid *de novo* assembly of both short- and long-read sequence data. The complete genomes revealed their highly similar genomic structures and a novel variant of SGI1-K underlying multidrug-resistant (MDR) patterns, including the presence of *bla_TEM-1b_*, which confers resistance to beta-lactams, including cephalosporins and *lnu*(F) which confers resistance to lincomycin and other lincosamides. In addition, the chromosomal mutations in the quinolone resistance-determining region (QRDR) were found at positions 83 (Ser83Phe) and 87 (Asp87Asn) of GyrA and at positions 57 (Thr57Ser) and 80 (Ser80Ile) of ParC suggesting high resistance to ciprofloxacin. We also compared SSSE-01 and SSSE-03 with publicly available complete genome data and revealed significant variations in SGI1-K genetic structures and variable relationships to antibiotic resistance. In comparison to the other isolates, SGI1-K of SSSE-01 and SSSE-03 had a relatively large deletion in the backbone, spanning from *S011* (*traG∆*) to *S027 (res*G), and the inversion of the IS*26-S044∆-yidY* segment. Their MDR region was characterized by the inversion of a large segment, including the *mer* operon and the relocation of *IntI1* and several resistance genes downstream of the IS*26-S044∆-yidY* segment. These structural changes were likely mediated by the recombination of IS*26*. The findings broaden our understanding of the possible evolution pathway of SGI1-K in fostering drug resistance, which may provide opportunities to control these MDR strains.

**IMPORTANCE:**

The emergence of ciprofloxacin-resistant (CIP^R^) *Salmonella* Kentucky ST198 globally has raised significant concerns. This study focuses on two poultry isolates from Thailand, revealing a distinct *Salmonella* genomic island 1 variant K (SGI1-K) genetic structure. Remarkably, multiple antibiotic resistance genes (ARGs) were identified within the SGI1-K as well as other locations in the chromosome, but not in plasmids. Comparing the SGI1-K genetic structures among global and even within-country isolates unveiled substantial variations. Intriguingly, certain isolates lacked ARGs within the SGI1-K, while others had ARGs relocated outside. The presence of chromosomal extended-spectrum *β*-lactamase (ESBL) genes and lincosamide resistance, *lnu*(F), gene, could potentially inform the choices of the treatment of CIP^R^*S*. Kentucky ST198 infections in humans. This study highlights the importance of understanding the diverse genetic structures of SGI1-K and emphasizes the role of animals and humans in the emergence of antimicrobial resistance.

## INTRODUCTION

Nontyphoidal *Salmonella enterica* (NTS) is one of the most important zoonotic pathogens that cause bacterial gastroenteritis in humans through the consumption of contaminated foods. With an estimated 93 million enteric illnesses and 155,000 fatalities per year, it is a significant public health problem worldwide (1, 2). *Salmonella* infections are mostly self-limiting, but antibacterial treatment may be indicated in the case of invasive infections or high-risk patients. Fluoroquinolones, particularly ciprofloxacin and third-generation cephalosporins, are the most frequent choices for the treatment of invasive NTS (3–5).

Emerging resistance to fluoroquinolones in *Salmonella* spp. has been recognized and the organism has been identified as one of 12 antibiotic-resistant “priority pathogens” with the greatest risks to human health (6, 7). In 2002, the first ciprofloxacin-resistant (CIP^R^) *S. enterica* serovar Kentucky (*S*. Kentucky) sequence type (ST) 198 was detected in French patients who traveled from Africa (8). Recently, *S*. Kentucky ST198 has spread across the world, especially in Northern Africa, Europe, and Southern Asia, and has become resistant to multiple classes of antimicrobial drugs, in addition to the fluoroquinolones which further complicates treatment (9). The strain has occasionally been reported as the most common serovar among NTS isolated from broilers in Thailand (10). *S*. Kentucky ST198 is not only often resistant to ciprofloxacin due to various mutations in the quinolone resistance-determining regions (QRDRs) of DNA gyrase (*gyr*A) and DNA topoisomerase IV (*par*C), but it is also multidrug-resistant (MDR) due to the presence of multiple antibiotic resistance genes in the chromosome, particularly in the *Salmonella* genomic island 1 (SGI1), mostly the variant SGI1-K (11, 12).

Originally, SGI1 was an integrative 42.4 kb chromosomal element first identified in the MDR *S. enterica* serovar Typhimurium phage type DT104 clone, which has caused an epidemic among humans and domestic animals (13–16). SGI1 consists of a 28 open reading frames (ORFs) backbone (*S001* to *S027* and *S044*) and an MDR region (17). The MDR region in the SGI1 island is a complex In4-type class 1 integron (named In104), originally clustering five antibiotic resistance genes (ARGs) that conferred resistance to ampicillin, chloramphenicol and florfenicol, streptomycin, spectinomycin, sulfonamides, and tetracycline. The acquisition of SGI1-encoding ARGs mostly appears to be result of homologous recombination or horizontal gene transfer (HGT) (18).

SGI1-K is a variant of SGI1 characterized by the deletion of *S005-S009* with the insertion of IS*1359*, a property that is also shared by SGI1-P and SGI1-Q. The 5′-end of *S044* of these SGI1 variants is also truncated by the insertion of IS*26*. SGI1-K typically has a complex multidrug resistance region, while the ones of SGI1-P and SGI1-Q are much simpler. It is generally believed that SGI1-K is a hotspot for the integration of ARGs into the chromosome of *S*. Kentucky. However, some complete genome sequence studies revealed the absence of any ARGs in SGI1-K of an MDR *S*. Kentucky ST198 strain (19–21).

Whole-genome sequencing (WGS) has been used for the characterization of antibiotic resistance elements in both genomes and plasmids of *Salmonella*. Although short-read next-generation sequencing (NGS) is commonly used to study microbial genomes for comparative analyses, the addition of long-read third-generation sequencing (TGS) data can yield more complete genomic structural variant information. To gain more insights into the and their relationship to drug resistance, we assembled the complete genome sequences of two epidemiologically related isolates of MDR *S*. Kentucky ST198, from poultry in Northeastern Thailand. We also conducted a phenotypic and genomic characterization focusing on antimicrobial resistance determinants. Both isolates harbored the same novel variant of SGI1-K. We additionally studied the variations of SGI1-K of other 28 high-quality complete genomes of *S*. Kentucky, available at NCBI. The comparison indicates a highly variable nature of SGI1-K genetic structures and variable genetic structural relationships to antimicrobial resistance. This finding provides insight into the spread and evolution of antibiotic resistance genes in CIP^R^ *S*. Kentucky ST 198.

## RESULTS

### Phenotypic characteristics of the studied isolates

The resistance to 10 antimicrobials was determined using the disk diffusion methods. Both strains exhibited similar levels of resistance to streptomycin, ampicillin, cefotaxime, norfloxacin, nalidixic acid, ciprofloxacin, and tetracycline, as shown in Table S1. However, SSSE-01 additionally showed intermediate resistance to amoxicillin-clavulanic acid. Therefore, both isolates were considered MDR including resistance to a third-generation cephalosporin and ciprofloxacin.

### The complete genomes of *S*. Kentucky SSSE-01 and SSSE-03

Both isolates were whole genome sequenced using both short- and long-read technologies on Illumina Nextseq500 and MinION Nanopore platforms, respectively. *De novo* hybrid assembly of both isolates was successfully performed using Unicycler V 0.4.8, with the sequence qualities summarized in Table S2. The hybrid assembly of both isolates resulted in four contigs each, comprising a complete-circular chromosome (4,870,588 bp and 4,869,812 bp for SSSE-01 and SSSE-03, respectively) and three circular-replicon col plasmids (4,018, 2,257, and 2,097 bp for both isolates). The genomes were annotated using the NCBI Prokaryotic Genome Annotation Pipeline and summarized in Tables S2 and S3. SSSE-01 and SSSE-03 were predicted to have 4,621 and 4,620 coding sequences (CDS), respectively.

### Multilocus sequence typing profile and phylogeny of the 28 isolates of *S*. Kentucky ST198

To investigate the phylogenetic relationships of our samples in the broader context of *S*. Kentucky diversity, we compiled complete genome sequences of *S*. Kentucky (*n* = 28) from the NCBI Genome database (https://www.ncbi.nlm.nih.gov/genome/, accessed on January 2023) that have annotation status of “Annotated by NCBI RefSeq.” Their metadata are provided in Table S4. Among these, eight samples were isolated from humans, while all others were isolated from animals or meat products. These isolates were from Spain (*n* = 10), Switzerland (*n* = 6), Israel (*n* = 2), USA (*n* = 2), China (*n* = 5), Canada (*n* = 1), and Indonesia (*n* = 2). The sequences of the samples from Spain, Israel, Switzerland, and PU131 from the US, and three samples from Anhui, China have been reported (18, 22–25). For all sequences, their multilocus sequence typing (MLST) genotype was identified using PubMLST (26). Two isolates (NZ_CP051346.1 and NZ_CP022500.1) were identified as belonging to ST152, while the ST of one Indonesian isolate (NZ_CP101647.1) could not be determined, and the other sequences were identified as ST198.

**Fig 1:**
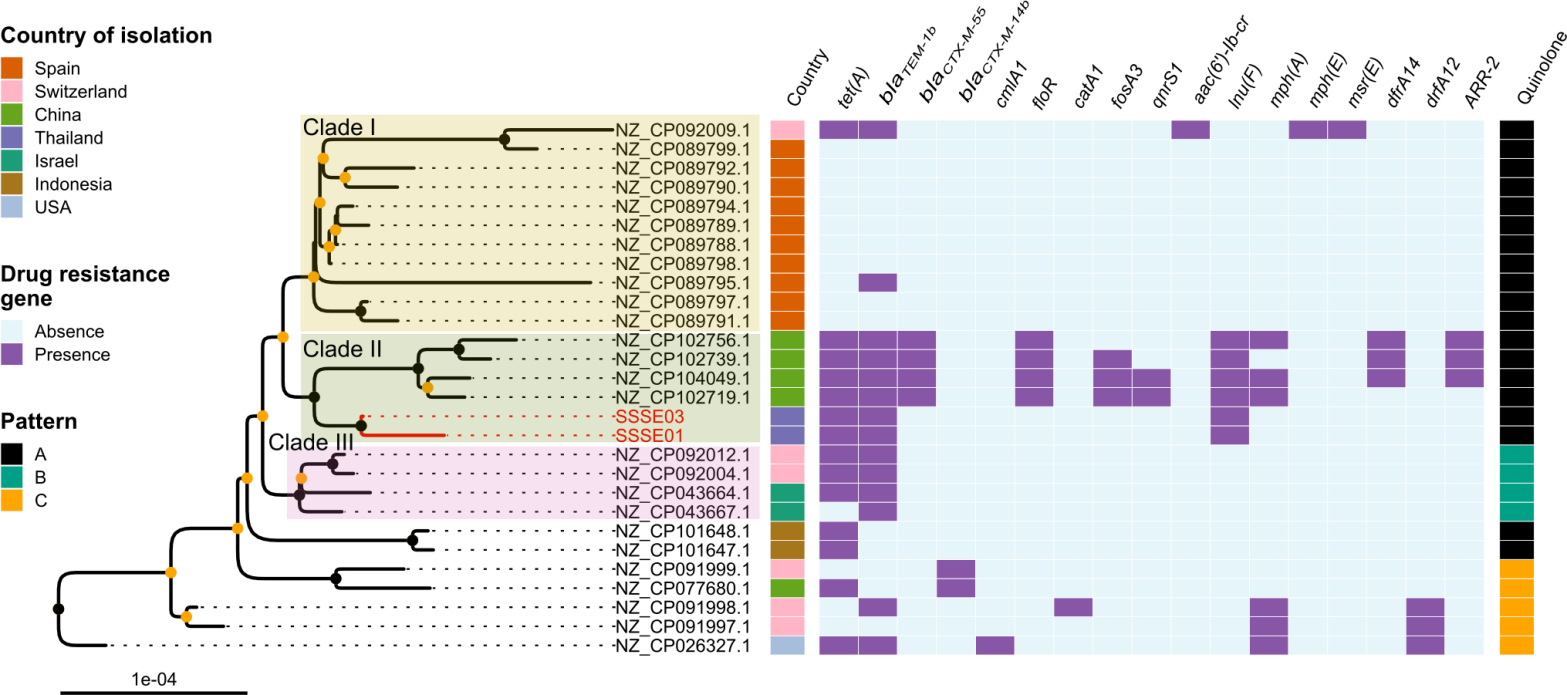
A maximum likelihood phylogeny of 28 isolates of *Salmonella* Kentucky ST198. The tree was inferred from a core genome SNP alignment (44,140 SNP sites) using IQ-TREE2 (28). The tree was rooted using the *S*. Kentucky ST152 (Accession numbers NZ_CP022500.1, NZ_CP051346.1) as outgroups (not shown here). The clade support values were computed using the ultrafast bootstrap (UFBoot) method (1,000 replicates), implemented in IQ-TREE2. For each internal node, black and orange points denote UFBoot values of 100 and >80, respectively. A scale bar indicates the branch length in the unit of substitutions per site. Panels from left to right show the country of isolation and the presence of some selected drug resistance genes. The rightmost panel shows patterns of amino acid changes in the quinolone resistance determining regions (QRDRs) within GyrA (the first two mutations) and ParC (the last two), including pattern A (black) representing the S83F, D87N, T57S, and S80I mutation, pattern B (green) representing the S83F, D87Y, T57S, and S80I mutation, and pattern C (orange) representing the S83F, D87G, T57S, and S80I.

Core genome single nucleotide polymorphisms (SNPs) for all sequences were identified by comparing their sequences to the *S*. Kentucky SSSE-01 sequence, using PhaME (27). Pairwise core genome SNP distances between SSSE-01 and SSSE-03 was 16 nucleotides. The pairwise SNP distances of the 28 sequences of *S*. Kentucky ST198 samples ranged from 1 to 112 nucleotides with the details shown in Table S5. The identified SNPs were used to construct a maximum likelihood phylogenetic tree. The tree shown in Fig. 1 contains only the isolates belonging to ST198 and the Indonesian isolate with unknown ST. The latter isolate was closely related to the other Indonesian ST198 isolates, with a pairwise SNP distance of seven nucleotides, and was, therefore, considered as belonging to the ST198 clade as well. Three isolates, PU131 from the US and two isolates (NZ_CP091997.1 and NZ_CP091998.1) from human urine from Switzerland, were early branching compared to the rest. There were three notable subclades. The first subclade included all samples from Spain and an isolate from Switzerland. The second subclade included four isolates from Anhui and Fujian in China, which were closely related to SSSE-01 and SSSE-03 from Thailand. The last subclade consisted of two isolates from Israel and two from Switzerland.

### Characterization of the gene content of the SGI1-K regions

**Fig 2:**
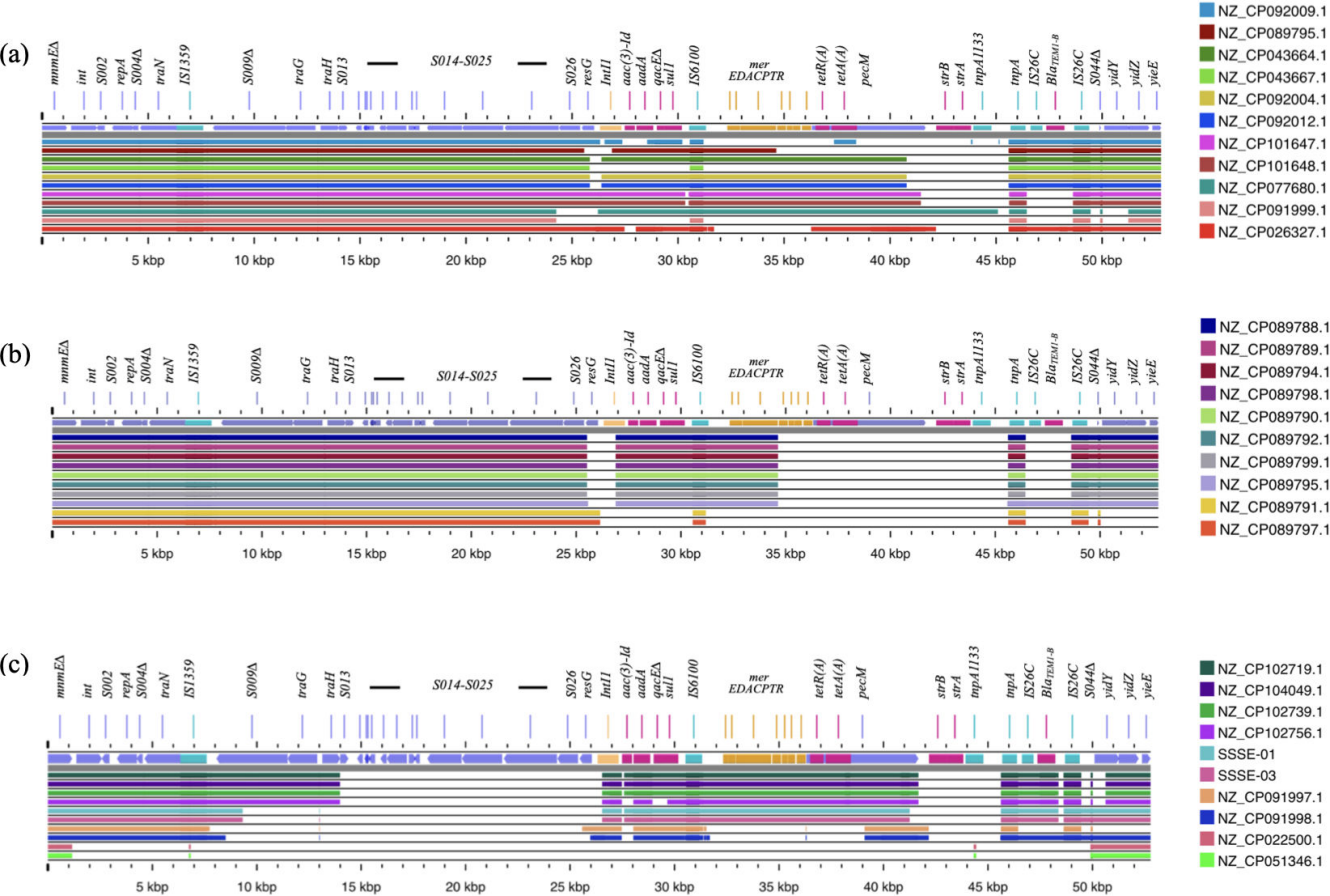
Genome content comparison of SGI1-K in 30 isolates to the 52,779-bp-long *S*. Kentucky SRC73 (AY463797.8) as reference. The presence of each DNA segment of each isolate was inferred from the result of BLAST using the reference sequence as the query. The results are categorized into three groups. Group (**a**) consists of isolates with long SGI1-K backbones. Group (**b**) includes the Spanish isolates. Group (**c**) includes isolates with short SGI1-K backbones, as well as two ST152 isolates, NZ_CP022500.1 and NZ_CP051346.1. The uppermost row of each figure indicates the positions of genes in the reference sequence AY463797.8. Genes are color-coded as follows: purple for CDS, yellow for *IntI1*, pink for drug resistance genes, and blue for insertion sequences.

To investigate the genetic structures of SGI1-K, we initially performed BLAST analysis of the complete genome sequences of all 30 *S*. Kentucky isolates included in this study using the 52,779-bp-long SGI1-K region of the SRC73 strain (Genbank Accession Number: AY463797.8) (29) as a query with the results shown in Fig. 2. The query sequence included the SGI1-K sequence as well as the flanking *mnmE∆* (also known as *trmE,* a part of *mnmE*) on the 5′-end and *yidY-yidZ-yieE∆* on the 3′-end, with the segment including *mnmE∆* to *yidY* being 50,841 bp long in SRC73.

The *S*. Kentucky ST152 isolates appeared to lack the SGI1-K sequence as they lacked the *S001-S027* backbone and *tnp6100*. The *mnmE∆* and *yidY* were still present, with the segment including them being only 4,025 bp long as shown in Fig. 2c.

All the other isolates contained SGI1-K, based on the presence of *mnmE∆* and some initial SGI1-K backbone genes. The *yidY* genes of some SGI1-K were partially deleted. The lengths of the DNA segments containing *mnmE∆* to *yidY* or *yidY∆* of 22 isolates ranged from 27,781 bp in SSSE-01 or SSSE-03 to 44,985 bp in NZ_CP101647.1 from Indonesia. The distance between *mnmE∆* to *yidY* of two isolates (NZ_CP091998.1 from Switzerland and NZ_CP089788.1 from Spain) was unusually long. The *yidY* gene was absent from the other five isolates as shown in Table S4.

The blasted segments of each isolate were then aligned to the SGI1-K region of the SRC73 strain. Among the 20 isolates with mostly intact SGI1-K backbone from *mnmE∆* to at least *S025*, six isolates had the complete backbone up to S027 (*resG*), similar to SRC73. These included the PU131 strain from US, an isolate from Switzerland (NZ_CP092009.1), a pair of isolates from Spain (NZ_CP089791.1 and NZ_CP089797.1, with a pairwise SNP distance of eight nucleotides), and a pair from Indonesia (NZ_CP101647.1 and NZ_CP101648.1, with a pairwise SNP distance of seven nucleotides).

In all, 12 isolates had partial deletions of *S027* (*resG*). This group included eight Spanish isolates (with an average pairwise distance of 28 nucleotides) and four isolates (with an average pairwise distance of 10 nucleotides) from Israel and Switzerland. It should be noted that even though NZ_CP089788.1 had a very long distance between *mnmE* to *yidY*, it still had the same SGI1-K genetic content as the other Spanish isolates, and had only three SNP different from NZ_CP089794.1, as shown in Table S5. In addition, a pair of isolates from China and Switzerland, with a pairwise SNP distance of 31 nucleotides, had similarly more deletion of *S026*, as shown in Fig. 2b.

Eight isolates, including SSSE-01 and SSSE-03, had extensive deletions in the SGI-1K backbone as shown in Fig. 2c. Four isolates from Anhui and Fujian, with an average pairwise distance of 14 nucleotides, had an identical backbone deletion from *S014* to *S027*. SSSE-01 and SSSE-03 exhibited an identical backbone deletion from the middle of *S011* (*traG*) to *S027* (*resG*). The last pair in this group were from Switzerland (NZ_CP091997.1 and NZ_CP091998.1) and had a pairwise SNP distance of only nine nucleotides. However, they had unusually long distances between *mnmE∆* to *yidY* and different profiles of deleted regions in SGI1-K. It is worth noting that a pair of isolates with similar gene contents in the SGI1-K region generally had a small average pairwise SNP distance.

### The genetic structures of the SGI1-K region in SSSE-01 and SSSE-03

**Fig 3:**
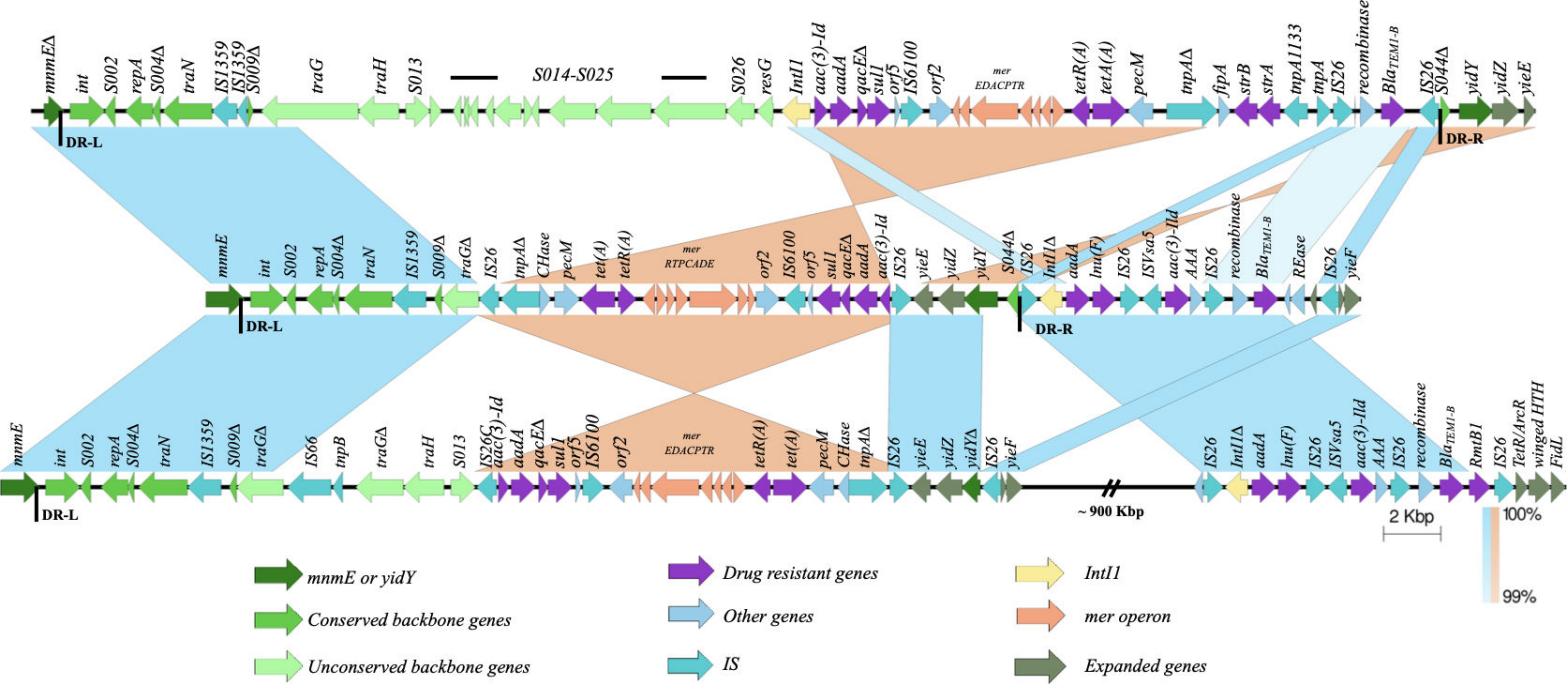
Comparison of SGI1-K regions of three isolates: SRC73 (AY463797.8) in the upper panel, SSSE-01 in the middle panel, and a representative isolate from Anhui, China (NZ_CP102719.1) in the lower panel.

The genetic structures of the SGI1-K region in SSSE-01 and SSSE-03 were identical, starting from the 5′-end with the DR-L sequence (5′-TTCTGTATTGGGAAGTAA-3′) in *mnmE* at the positions 4,866,037-54 and 4,865,261-78 of SSSE-01 and SSSE-03, respectively, in the complementary sequence. Compared to the original SGI1-K, the ones in SSSE-01 and SSSE-03 were substantially shorter, mainly due to the deletion of approximately 20 kb of nucleotides (from the position in AY463797.8 at 8,067–27,330 bp). This deletion included a part of *S011* (*traG*), as well as *S012-S027* and *IntI1*, accounting for almost 30% of the *mnmE*∆ to *IntI1* segment. The *traG*∆ was immediately followed by a copy of IS*26*, followed by a part of the segment containing ARGs of SRC73 including *aac (3)-Id, aadA7, qacE∆, sul1*, IS*6100*, *mer* operon, *tetR(A), tet(A), pecM,* and *tnpA*∆ but in inverted orientation, and finally followed by another copy of IS*26*. The segment was likely to be inverted by the recombination of IS*26*. Immediately downstream, the segment containing *yieE-yidZ-yidY-S044∆* was also inverted compared to the original SGI1-K, as shown in Fig. 3. This resulted in the inversion of the DR-R in *yidY* to 5′-TTCTGTATTGGTAAGTAA-3′ at positions 4,839,421-38 and 4,838,645-62 of SSSE-01 and SSSE-03, respectively. The *yieE-yidZ-yidY-S044∆* segment, which is generally considered the end of SGI1-K, was immediately followed downstream by a multi-resistance region (MRR), comprising three IS*26*-flanking segments: *IntI1-aadA-lnu(F*), IS*4-aac (3)-IId-AAA*, and recombinase-*bla_TEM-1b_*, respectively. Only the third segment was present partially in SRC73. The last IS*26* was followed by *yieF*, which is normally found after *yidY-yidZ- yieE* in ST198. The SGI1-K region of SSSE-01 and SSSE-03 had a total of six copies of IS26, instead of two in the original SGI1-K (Fig. 3).

The four isolates from Anhui and Fujian in China were the most closely related to SSSE-01 and SSSE-03. The SGI1-K region of the isolate NZ_CP102719.1 (AH19MCS1) (25) is exemplified in Fig. 3. It had less deletion of the backbone. They contained the same ARG-containing segment as SSSE-01 and SSSE-03, which retained the same orientation as SRC73, though. The ARG-containing segment was followed by a segment containing *yieE-yidZ-yidY∆*, with a part of *yidY* and *S044∆* being deleted. This segment was immediately followed by *yieF*. Interestingly, the Chinese isolates also contained an MRR, almost the same as SSSE-01 and SSSE-03, residing 900 kbp downstream from the *yieF*, as recently reported.

A complete sequence of SGI1-K of another isolate of *S*. Kentucky CIP^R^ ST198 isolates in Thailand has been recently reported. The isolate contained the same truncated backbone and the inverted *yieE-yidZ-yidY-S044∆* segment, similar to SSSE-01 and SSSE-03. The segment containing the *mer* operon was inverted compared to SSSE-01 and SSSE-03, and it lacked *Intl-aadA-lnu(F*) and IS*4-aac (3)-IId-AAA* segments (30).

### The genetic structures of SGI1-K of other *S*. Kentucky ST198

**Fig 4:**
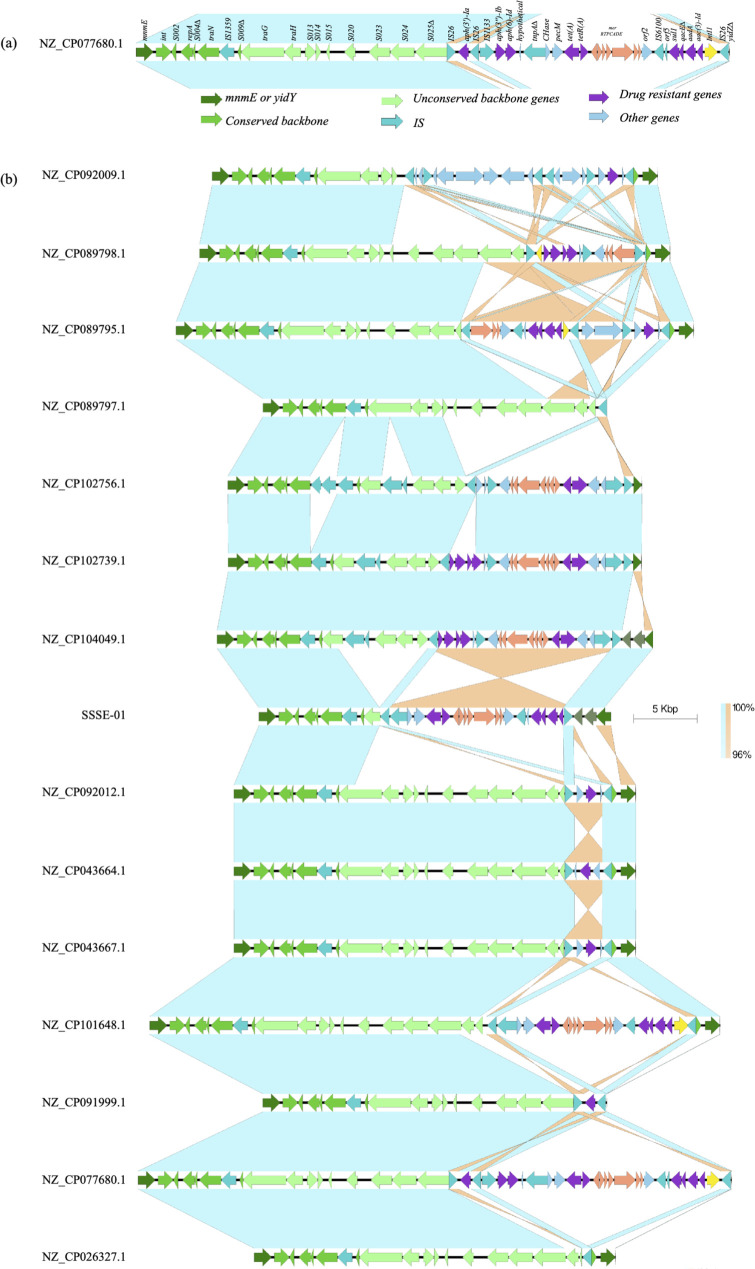
Comparison of the genetic structures of SGI1-K of several representative samples of *S*. Kentucky ST198. (**a**) The SGI1-K of NZ_CP077680.1, which was the longest among this group, is shown with labeled genes. (**b**) The SGI1-K of several representative isolates from *mnmE* to *yidY*. In cases where *yidY* is missing, the regions were shown to the last IS*26*. A group of isolates with very similar genetic structures is shown only once. The figure is ordered to correspond to the order in the phylogenetic tree in Fig. 2. The last isolate, NZ_CP026327.1, was PU131.

**Fig 5:**
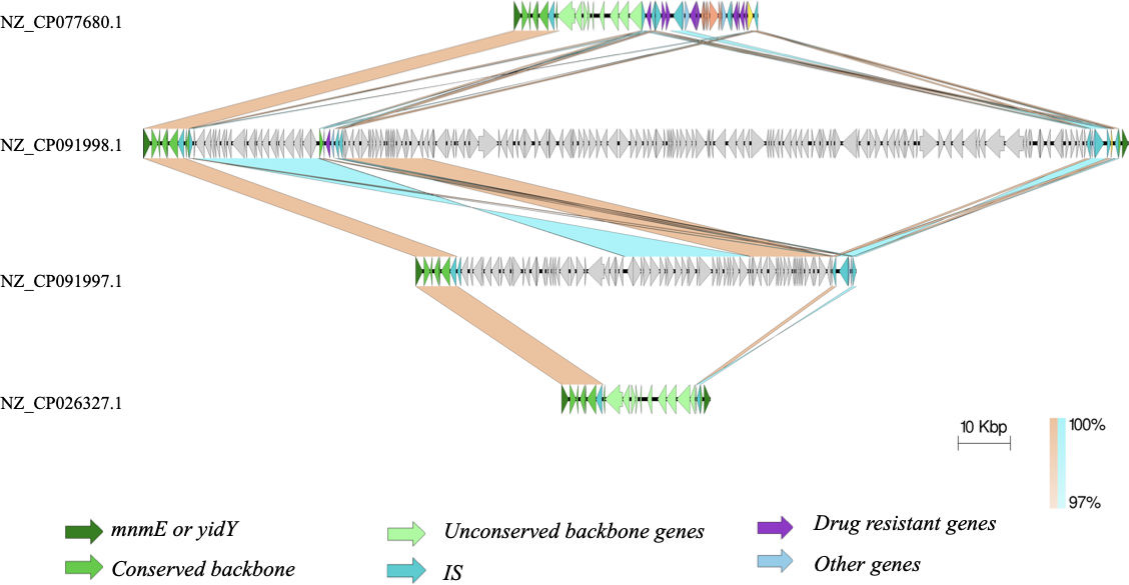
Comparison of the genetic structures of SGI1-K in two early branching isolates from Switzerland, NZ_CP091997.1 and NZ_CP091998.1, compared with NZ_CP077680.1 and PU131 (NZ_CP026327.1). In both Swiss isolates, SGI1-Ks were fragmented. Specifically, the 5′ end of their SGI1-K backbones remained conserved but truncated, while 3′-ends were scattered to distant sites in the chromosome. NZ_CP077680.1, shown in Fig. 4a, was included for comparison.

Comparison of the genetic structures of the SGI1-K in other S. Kentucky ST198 revealed considerable variations, the representatives of which are shown in Fig. 4 and 5. Interestingly, the order of the core genes from *S001-S027* was mostly consistent among all isolates, although there were variable deletions involving *S011* to *S027*. All isolates still retained at least the segment between *S001* and *traG* (*S011*). However, the regions between *S027* to *S044∆*, which typically contained ARGs and the *mer* operon, exhibited high variability, including both insertions/deletions (in/del) and inversions. These variable segments were often flanked, and therefore, likely mediated by recombination of IS*26*. In addition, the ARGs themselves were not necessarily located within the conventional boundary of SGI1-K, upstream to *S044∆-yidY*. Instead, they were found in other genomic locations.

Manual comparison of SGI1-K revealed that three isolates, PU131, and two Spanish isolates (NZ_CP089791.1 and NZ_CP089797.1), did not contain any ARGs or *mer* in the SGI1-K region, although the backbones were mostly intact. In PU131, the segment between *S027* and *S044* was completely absent, likely due to recombination of IS*26*. However, it contained 10 ARGs that had been relocated into multiple sites elsewhere in the genome, as previously reported (22). The two Spanish isolates had only a single ARG, *aac(6′)-Iaa*, in their chromosome but not in SGI1-K. This gene, which acetylates tobramycin, kanamycin, and amikacin effectively but not gentamicin (31)*,* was present in all studied ST198 strains and was originally identified in the chromosome of *S*. Typhimurium LT2 strain. Both isolates also lacked the *IS26-S044-yidY* segment.

Some SGI1-K elements contained only a single ARG. The four isolates belonging to the Israel/Switzerland subclade had nearly intact backbones but lacked the *mer* operon and contained only a single ARG, *bla_TEM-1_*, flanked by two IS*26* and located between *S027* and *S044∆-yidY* in SGI1-K. The other ARGs typically found in SGI1-K [e.g., *aadA7, aac (3)-Id, sul1,* and *tet(A*)] were previously shown to be relocated into *rbsK* (26). Two other isolates from Switzerland (NZ_CP091999.1 and NZ_CP092009.1) also had only one ARG in the SGI1-K region. NZ_CP091999.1 harbored *aph(3′’-Ia* and lacked the IS*26-S044∆-yidY* segment. NZ_CP092009.1 had a highly rearranged SGI-1K between *S014* and the *IS26-S044∆-yidY* segment, and it only harbored only *bla_TEM-1_* in SGI1-K.

All nine Asian isolates had complex ARG-containing regions in SGI1-K, which were partially homologous to the one in SRC73. A Chinese isolate, NZ_CP077680.1, had the longest ARG segment, including *Intl1*, *aac (3)-Id, aadA7, qacE∆, sul1*, IS*6100*, *mer* operon, *tetR(A), tet(A), pecM* and *tnpA∆*, *aph (6)-Id, aph(3″)-Ib,* IS*1133,* IS*26*, and *aph(3′)-Ia* in an inverted orientation as shown in Fig. 4b. The homologous segments of both Indonesian isolates (NZ_CP101647.1 and NZ_CP101648.1) were shorter, with truncation after *tnpA∆* by IS*26*, also in the inverted orientation. The homologous segment of SSSE-01 and SSSE-03, also in the inverted orientation, was further truncated by another IS*26* inserted between *IntI1* and *aac (3)-Id*. The Anhui and Fujian isolates had a segment with a similar length to the Thai isolates but with an orientation similar to SRC73 as shown in Fig. 4. These similarities suggested that the evolution of the ARGs region of SGI-1K may have been mediated by IS*26* insertions and homologous recombination.

The Spanish isolates exhibited several patterns of the SGI1-K element. Two of them had no ARGs in SGI-1K, as described above. Six isolates had the same intact backbone up to *S026* followed by IS*26,* a segment containing *IntI1*-to-*merEDA*, another copy of IS*26*, and finally *S044∆-yidY*. These isolates retained some ARGs in the original SGI1-K, namely *aac (3)-Id, aadA7, qacE∆, sul1*. In another isolate, NZ_CP089795.1, an additional IS*26* was inserted in *S025* with the inversion of the segment between this IS*26* and the one upstream of the *mer* genes. This resulted in the presence of *S026* and *S025* sequences downstream of the ARGs as shown in Fig. 4a. In the last Spanish isolate, NZ_CP089788.1, SGI1-K was scattered in the chromosome.

The *yidY* gene was absent in five isolates, as shown in Table S4. It was partially deleted from all four isolates from Anhui and Fujian, and this deletion was accompanied by the loss of the DR-R segment. The absence of the DNA segment was recently reported in the majority of *S*. Kentucky ST198 isolates from China (25). Among the other isolates, the intact *yidY* genes were identified mostly in the typical IS*26-S044∆-yidY* segment oriented similarly to SRC73, except the two Thai isolates, which were in reversed orientation. A variant of the *IS26-S044∆-yidY* segment was identified in seven isolates from Spain, represented by NZ_CP089798.1 and NZ_CP089795.1 in Fig. 4a.

The *bla_TEM-1_* gene was identified in 14 isolates. The gene was in a Tn2-like structure within the typical boundary of SGI1-K in six isolates, including the Israel/Switzerland clade, NZ_CP089795.1 from Spain, and NZ_CP092009.1 from Switzerland. However, the gene was outside the typical SGI1-K boundary in the four isolates from Anhui and Fujian, two isolates from Thailand, PU131 and NZ_CP091998.1.

*bla_CTX-M-14b_* was identified in two isolates from China and Switzerland. It was previously shown to be inserted in a 2,854 bp fragment inserted in the chromosomal region containing Type 6 Secretion System genes, downstream of the *hcp1* gene (position 3,701,330 of reference genome NZ_CP126327.1). The fragment is flanked on one side by an insertion sequence *ISEcp1* of the *IS1380* family (32, 33). The 2,854 bp fragment was absent on the chromosome of the non-*bla_CTX-M-14b_ S*. Kentucky isolates. *S*. Kentucky harboring *bla_CTX-M-14b_* has been reported several times from China (34).

The *bla_CTX-M-55_* was present in the chromosome but not in the SGI1-K region in all four isolates from Anhui and Fujian. A recent study in Anhui identified *bla_CTX-M-55_* in all 10 *S*. Kentucky isolates (25).

### Antibiotic resistance gene profiles of SSSE-01 and SSSE-03

*S*. Kentucky ST198 strains have become highly resistant to ciprofloxacin by accumulating various combinations of mutations in the QRDRs of *gyrA*, encoding a subunit of DNA gyrase and *par*C, encoding a subunit of DNA topoisomerase IV (15). All 28 isolates had Ser83Phe substitution in GyrA. All three mutations affecting Asp87 were present among the 28 isolates. All the five early branching isolates in the phylogenetic tree had Asp87Gly while Asp87Tyr was unique to the four-isolate clade from Israel and Switzerland. The other 19 isolates, including SSSE-01 and SSSE-03, had Asp87Asn. All isolates had the quinolone resistance associated with mutations of both Thr57Ser and Ser80Ile in ParC.

ResFinder identified various ARGs, ranging from 1 to 18 per isolate as summarized in Table S6 and Fig. 1. The *aac(6′)-Iaa* gene was identified in all isolates, similar to a previous report (35), while *aadA7, aac (3)-Id*, and *sul*1 were present in the majority. The *tet(A*) and *bla_TEM-1B_* genes were identified in about half of the isolates. SSSE-01 and SSSE-03 additionally harbor *aadA17* and *lnu(F*). The isolates from Anhui and Fujian harbored the most numbers of ARGs, including almost all of the mentioned ARGs. They, as a group, uniquely harbored *bla_CTX-M-55_, rmtB,* and *floR*. Some of them also uniquely harbored *fosA3, qnrS1, aac (3)-Id, aac (3)-IV*, and *aph (4)-Ia*. They also harbored *aadA17, aac (3)-Iid,* and *lnu(F*) similar to SSSE-01 and SSSE-03, and *aph(3′)-Ia* similar to other isolates. A single colistin resistance gene, *mcr-1.1* (chromosomal position at nt 4,160,060–4,161,685), was identified in the sample NZ_CP077680.1, isolated in 2020 from chicken at an unspecified location in China (16).

### Plasmids of *S*. Kentucky strains

Both SSSE-01 and SSSE-03 contained three plasmids, with the same sizes of 4,018 bp, 2,257 bp, and 2,097 bp, and nearly identical sequences in both isolates as summarized in Table S3. These plasmids did not contain any ARGs. PlasmidFinder identified the plasmids pSSSE01a and pSSSE03a as having a similar replicon (91.6%) to Col(pHAD28) (accession number KU674895), as shown in Fig. S1. The replicons of plasmids pSSSE01b and pSSSE03b were similar (88.1%) to Col(MP18) (accession number NC013652). The replicons of plasmids pSSSE01c and pSSSE03c were highly similar (98.96%) to ColpVC (accession number JX133088), which was associated with various mobile genetic elements and is known to carry multiple antibiotic resistance genes.

## DISCUSSION

*S*. Kentucky is commonly reported from poultry worldwide (32, 36–39). The serovar is polyphyletic, consisting of strains belonging to several genotypes, including ST152, ST198, ST314, etc. (33). Human diseases caused by *S*. Kentucky are relatively uncommon, and to our knowledge, there have been no reports of human diseases caused by *S*. Kentucky in Thailand yet. However, salmonellosis caused by ST198 has been reported in several European countries, occasionally with travel histories to Africa and Asia (8, 40). Recent studies have also indicated the increased public health importance of *S*. Kentucky (41). For example, *S*. Kentucky was identified in 48% of NTS isolated from hospitalized diarrheal cases around Delhi, India (42). ST198, in particular, poses significant public health implications, while ST152 does not raise the same level of concern. Overall, these findings underscore the emerging importance of *S*. Kentucky as a potential health threat.

A clade of *S*. Kentucky ST198, which exhibits resistance to ciprofloxacin (CIP^R^ ST198), is of particular concern, as the drug is commonly used to treat salmonellosis. The clade also usually harbors extended-spectrum beta-lactamase (ESBL) genes, making it resistant to common alternative drugs, third-generation cephalosporins (17, 43). In this study, we reported the complete genome sequences of two isolates of CIP^R^*S*. Kentucky ST198, which carried an ESBL gene, *bla_TEM-1b_*, and also *lnu*(F), which made them resistant to cephalosporins and lincosamide. Therefore, these strains would be difficult to treat if necessary.

While ARGs in Enterobacteriaceae, including *Salmonella*, are often located in plasmids, which facilitate their transmission across several species and genotypes, resistance to ciprofloxacin the CIP^R^ ST198 clade is due to the mutations in chromosomal target genes, *gyrA* and *parC* (11, 12). Moreover, several ESBL genes, including *bla_TEM-1b_, bla_CTX-M-14_,* and *bla_CTX-M-55_*, were identified in the chromosome, among which only *bla_TEM-1b_* was identified in SGI1-K (43–46). Therefore, focusing surveillance on the transmission of the CIP^R^ ST198 clade would be useful in controlling the spread of the MDR strains.

In this study, we also identified a previously unreported isolate from China, NZ_CP077680.1, harboring intact *mcr-1.1*, which confers resistance to colistin. The presence of *mcr* may lead to the more challenging treatment of the strain in the future (16).

SGI1 is known as a hotspot for the integration of ARGs in many species of Enterobacteriaceae (13–16). SGI1-K was initially discovered in *S*. Typhimurium and might have transferred to *S*. Kentucky ST198 shortly before 1990 (17). Previous studies using NGS short-read data have shown that the region in SGI1-K between *resG* (*S027*) and *S044∆* is the primary site in the *Salmonella* genome where most ARGs are located (9). However, by analyzing the complete genome sequences, mostly from the hybrid assembly, we demonstrated significant variation in SGI1-K, which was previously sporadically reported (17). Some isolates from Europe and the USA completely lost the ARG-containing segment from SGI1-K or retained only a single ARG. By contrast, isolates from Asia mostly retained some ARGs originally reported in SGI1-K of the SRC73 strain. In addition, both groups occasionally carried more ARGs in other chromosomal regions. The role of SGI1-K in the formation of the multidrug resistance gene regions outside itself is not clear, as there is evidence suggesting that the integration of some ESBL genes to the chromosome may not require the SGI1-K region (47).

The substantial genetic structural variations of SGI1-K in CIP^R^ ST198 are not limited to the ARG-containing region. While the order of the backbone genes of SGI1-K is mostly conserved, with the segment from *mnmE* to *S010* always present, the rest of the backbone can be deleted or inverted, primarily mediated by transposition and homologous recombination of IS*26*. The 3′-end of SGI1-K exhibited high variability. The IS*26-S044*∆-*yid*Y segment is not always present; it can be inverted or completely absent. Even when the IS*26-S044*∆-*yidY* segment was present, ARGs may not necessarily be located in the segment between it and *mnmE*. Further study is needed to understand how the evolution of the SGI1-K backbone affects its function as a hotspot of integration of ARGs.

In conclusion, even with a limited number of samples included in this study, their complete genomes revealed vast genetic structural variations of SGI1-K among CIP^R^ *S*. Kentucky ST198. Resistance to third-generation cephalosporins was mediated by a variety of *bla_TEM1_ or bla_CTX_* genes located in many sites in the chromosome including SGI1-K. The exact location of them may provide a method for recognizing various circulating clades of the bacteria. The identification of *lnu*(F) and *mcr-1.1* signifies the existence of lincosamides and colistin-resistant clones which require close monitoring.

## MATERIALS AND METHODS

### Collection of *S*. Kentucky strains

Two *S*. Kentucky isolates, SSSE-01 and SSSE-03, were isolated from a chicken slaughterhouse in Mukdahan, a province in Northeast Thailand bordering the Lao People’s Democratic Republic as a part of a surveillance project in 2016. The protocol of the sample collection was approved by the Faculty of Veterinary Science-Animal Care and Use Committee at Mahidol University, under protocol number MUVS-2016-09-38. Both isolates were identified as serovar Kentucky using a serological test at the Ministry of Science in Thailand. WGS of both were done with Illumina NextSeq 500 using paired-end reads. The sequencing data has been deposited under the PRJNA841788 project, with accession numbers AAEXAW010000000 and AAEXAP010000000. The serovar designation and MLST were confirmed *in silico* using the Enterobase database (https://enterobase.warwick.ac.uk/species/index/senterica). In addition, complete genome sequences of 28 *S*. Kentucky isolates collected were retrieved from NCBI (www.ncbi.com) as shown in Table S4. These isolates were confirmed to be *S. enterica* by the analysis of WGS and the sequence types were identified using the PubMLST database (26).

### Antimicrobial susceptibility assay

Antimicrobial susceptibility assay was conducted using the disk diffusion method of the Kirby-Bauer technique, following the protocols of the Clinical and Laboratory Standards Institute (48). Ten antimicrobials (Oxoid Ltd., Basingstoke, UK) were tested: ampicillin (AMP, 30 µg), amoxicillin-clavulanic acid (AMC, 20 µg/10 µg), cefotaxime (CTX, 30 µg), streptomycin (S, 10 µg), tetracycline (TET, 30 µg), ciprofloxacin (CIP, 5 µg), norfloxacin (NOR, 10 µg), nalidixic acid (NA, 30 µg), (30 µg), trimethoprim-sulfamethoxazole (SXT, 1.25 µg/23.75 µg), and chloramphenicol (CHL, 30 µg). The inhibition zones were measured and interpreted using the CLSI guideline (48). *Escherichia coli* ATCC 25922 was used as control. Multidrug resistance (MDR) was defined as antimicrobial resistance to at least three classes of antibiotics.

### Genomic DNA extraction and Illumina sequencing

*S*. Kentucky isolates were cultured on trypticase soy agar (TSA) at 37°C for 24 h. Genomic DNA (gDNA) was extracted using the QIAmp DNA mini kit (Qiagen, Hilden, Germany). The quality and quantity of DNA were assessed using Nanodrop DenoVix for absorbance value and the Qubit 3.0 fluorometer for double-strand DNA quantity. Purified gDNAs of SSSE-01 and SSSE-03 were sequenced at the Advanced Genomic Technologies Cluster New York State Department of Health/Wadsworth Center, NY by Illumina NextSeq500 instrument, using the Nextera XT library preparation protocol and the NextSeq 500/550 Mid Output Kit v2.

### Oxford nanopore library preparation and sequencing

Genomic DNA samples that met the following criteria were used for library construction: (i) A260/280 between 1.8 and 1.9, and (ii) A260/230 between 2.0 and 2.2. Approximately 400 ng of total input DNA was used for each flow cell (FC). Electrophoresis was performed to separate and determine the sizes of linear DNA fragments.

The gDNA samples underwent ligation (SQK-LSK110) following the library preparation protocol provided by Oxford Nanopore Technologies (ONT). The libraries were then sequenced using qualified FLO-MIN106 flow cells (R9.4.1, with an active pore number >800) on the MinION (ONT, Oxford, UK) for approximately 48 h. Real-time basecalling was performed using Guppy, with a modified basecalling model for 6 mA dam/5 mC dcm and CpG, which was integrated into the MinKNOW software v3.5.40 installed on MinION.

### *De novo* hybrid assembly

The fastq files containing the short-read data from the Illumina Nextseq 500 sequencing and the long-read MinIon Nanopore sequencing were assessed using FastQC [ver. 0.11.5 (49)] and Nanoplot tool [ver. 1.28.2 (50)], respectively. Trim Galore [ver. 0.6.7 (51)] was utilized to remove low-quality reads and adapters from the short reads, while Porechop [ver 0.2.4 (52)] was employed to remove adapters from the long reads and Trimmed reads with the minimum length of 1,000 bp was used for subsequent assembly using Filtlong (ver. 0.2.1 (53). Subsequently, both the short and long reads were combined for hybrid *de novo* assembly using Unicycler version 0.4.8 (54).

### Genomic characterization

The genomic assemblies of two isolates were uploaded to the NCBI server for genome annotation using the Prokaryotic Genome Annotation Pipeline (PGAP, https://www.ncbi.nlm.nih.gov/genome/annotation_prok). MLST was done using PubMLST online tools with *Salmonella* spp. as the organisms of interest. To identify antimicrobial resistance (AMR) genes, ResFinder V. 4.1 (55) was used with a minimum identity of 95% and a minimum length of 60%. Plasmid identification was performed using PlasmidFinder (56), with a minimum identity of 80% and a minimum length of 60%.

### Mapping of gene contents of SGI1-K

A database comprising all ORFs from SGI1-K of *S*. Kentucky SRC73 (AY463797.8) was built specifically for this study. To align and display the location of SGI1-K, BLAST analysis of annotated genes with 0.0001 of e-value cutoff was performed using the Proksee tool (https://proksee.ca/).

### Multiple sequence alignment and phylogenetic tree analysis

To provide context for our newly assembled sequences, we searched in the NCBI database to identify previously reported sequences of *S*. Kentucky. We retrieved a total of 30 genome sequences that were designated as Annotated by NCBI RefSeq by NCBI and were accessible in January 2023. Then, PhaME (27) was used for sequence alignment and core genome SNP identification using the SSSE-01 as a reference sequence, because of its high read depths and geographic proximity to many unreported samples, which are mostly in Asia. The resulting core genome SNP alignment was then used to compute pairwise SNP difference using snp-dists (57), and maximum likelihood phylogenetic reconstruction using IQ-TREE 2 (28). A comparison of SGI1-K was done by Easyfig (58).

## Data Availability

The NGS data of SSSE-01 and SSSE-03 are available under the PRJNA183850 project, accession numbers AAEXAW010000000 and AAEXAP010000000, respectively. The TGS data of SSSE-01 and SSSE-03 are available under PRJNA841788. In addition, the assembled contigs were deposited in Genbank under the accession numbers CP097849-CP097852 for SSSE-01 and CP097853-CP097856 for SSSE-03.

## SUPPLEMENTAL MATERIAL

Supplemental material spectrum.03994-23-s0001.docx Tables S1 to S6; Fig. S1.

ASM does not own the copyrights to Supplemental Material that may be linked to, or accessed through, an article. The authors have granted ASM a non-exclusive, world-wide license to publish the Supplemental Material files. Please contact the corresponding author directly for reuse.
