# Supplementary material for: A novel variant in Salmonella genomic island 1 of multidrug-resistant Salmonella enterica serovar Kentucky ST198
Source: Microbiol Spectr. 2024 Apr 30;12(6):e03994-23. doi: 10.1128/spectrum.03994-23 (PMC11237444; doi:10.1128/spectrum.03994-23)
Supplement: Supplemental material — Tables S1 to S6; Fig. S1. [file spectrum.03994-23-s0001.docx]

**Supplementary Information**

**A novel variant in *Salmonella* genomic island 1 of multidrug-resistant *Salmonella* *enterica* serovar Kentucky ST198**

**Rattanaporn Intuy^1^, Sirirak Supa-Amornkul^1,2^, Bharkbhoom Jaemsai^1^, Wuthiwat Ruangchai^1^, Witthawat Wiriyarat^3^, Soraya Chaturongakul^1,4^, Prasit Palittapongarnpim^1*^**

^1^Professor Pornchai Matangkasombut Center for Microbial Genomics (CENMIG), Department of Microbiology, Faculty of Science, Mahidol University, Bangkok, Thailand

^2^Department of Oral Microbiology, Faculty of Dentistry, Mahidol University, Bangkok, Thailand

^3^Department of Pre-Clinical and Applied Animal Science, Faculty of Veterinary Science, Mahidol University, Thailand

^4^Molecular Medical Biosciences Cluster, Institute of Molecular Biosciences, Mahidol University, Bangkok, Thailand

^*^Corresponding author: Prasit Palittapongarnpim, Professor Pornchai Matangkasombut Center for Microbial Genomics (CENMIG), Department of Microbiology, Faculty of Science, Mahidol University, Rama 6 Road, Bangkok, 10400 Thailand. [prasit.pal@mahidol.ac.th](mailto:prasit.pal@mahidol.ac.th)

**Supplementary Table**

**Table S1**. Antibiotic susceptibility profiles of *S.* Kentucky SSSE-01 and SSSE-03 using disk diffusion methods.

| **Class of Antibiotic** | **Drug** | **ID** | **Dose (µg)** | **Zone of inhibition (mm)*** | | **Interpretation**** | |
| --- | --- | --- | --- | --- | --- | --- | --- |
|  |  |  |  | **SSSE-01** | **SSSE-03** | **SSSE-01** | **SSSE-03** |
| **Aminoglycosides** | streptomycin | S10 | 10 | 0 | 0 | R | R |
| **Beta-lactams** | ampicillin  cefotaxime | AMP10  CTX30 | 10  30 | 0 | 0 | R | R |
|  | amoxicillin-clavulanic acid | AMC | 20/10 | 17 | 20.47 | I | S |
| **Quinolones** | ciprofloxacin  nalidixic acid  norfloxacin | CIP5  NA30  NOR10 | 5  30  10 | 10.29  0  8.12 | 10.82  0  0 | R  R  R | R  R  R |
| **Folate pathway blockers** | trimethoprim-sulfamethoxazole | SXT25 | 1.25/23.75 | 26.31 | 19.30 | S | S |
| **Phenicol** | chloramphenicol | C30 | 30 | 26.60 | 21.40 | S | S |
| **Tetracycline** | tetracycline | TE30 | 30 | 0 | 0 | R | R |

* The sizes of inhibition zones were averaged from three replicates. A value of 0 indicates the absence of any inhibition zone.

** I for intermediate, R for resistance, and S for sensitive

*** AMC, contains 20 µg of amoxicillin and 10 µg of clavulanate, resulting in a total dosage of 30 µg. SXT25, contains 1.25 µg of trimethoprim and 23.75 µg of sulfamethoxazole, with a total dosage of 25 µg.

**Table S2.** The results of Nanopore sequencing and *de novo* hybrid genome assembly of SSSE-01 and SSSE-03.

| **Data** | **SSSE-01** | **SSSE-03** |
| --- | --- | --- |
| **Total read bases (bp)** | 1.42 Gb | 1.06 Gb |
| **Number of long reads** | 195,028 | 105,628 |
| **Median read length** | 2,517 | 3,537 |
| **Mean read length** | 7,296 | 10,040 |
| **Coverage (x)** | 292 | 218 |
| **N50 (bp)** | 21,537 | 26,396 |
| **Assembled Genome size (bp)** | 4,870,588 | 4,869,812 |
| **Number of CDS (total)** | 4,621 | 4,620 |
| **Numbers of t-RNA genes** | 84 | 84 |
| **Numbers of prophage CDS** | 120 | 117 |
| **rRNAs (5s, 16s, 23s)** | 8, 7, 7 | 8, 7, 7 |
| **Non-coding RNA** | 14 | 14 |
| **GC contents (%)** | 52.2 | 52.2 |
| **Plasmid sizes (bp)** | 4,018 | 4,018 |
|  | 2,257 | 2,257 |
|  | 2,097 | 2,097 |

**Table S3.** Plasmids in *S.* Kentucky strain SSSE-01 and SSSE-03 are identical.

| **Plasmids** | **Most similar Plasmid replicons** | **%**  **Similarity** | **Plasmid Sizes (kb)** | **Accession numbers of the reference plasmids** |
| --- | --- | --- | --- | --- |
| **SSSE-01** |  |  |  |  |
| pSSSE01a | Col(pHAD28) | 91.6 | 4,018 | KU674895 |
| pSSSE01b | Col(MP18) | 88.08 | 2,257 | NC013652 |
| pSSSE01c | ColpVC | 98.96 | 2,097 | JX133088 |
| **SSSE-03** |  |  |  |  |
| pSSSE03a | Col(pHAD28) | 91.6 | 4,018 | KU674895 |
| pSSSE03b | Col(MP18) | 88.08 | 2,257 | NC013652 |
| pSSSE03c | ColpVC | 98.96 | 2,097 | JX133088 |

**Table S4**. The list of the complete genome sequences of *S.* Kentucky isolates used in this study.

| **No.** | **Strains** | **Accession number** | **Size of genome** | **Place of isolation** | **Year of isolation** | **Source of isolation** | **Calculated sizes of SGI1-K**  ***mnmE* and *yidY*** | **Reference** |
| --- | --- | --- | --- | --- | --- | --- | --- | --- |
| 1 | SSSE-01 | NZ_CP097849 | 4,870,588 | Thailand | 2016 | Chicken | 27,781 | This study |
| 2 | SSSE-03 | NZ_CP097853 | 4,869,812 | Thailand | 2016 | Chicken | 27,781 | This study |
| 3 | PU131 | NZ_CP026327.1 | 4,900,326 | USA: WA | 2013 | Human feces | 28,497 | (18) |
| 4 | 161365 | NZ_CP043664.1 | 4,889,798 | Israel | 2015 | Animal | 31,710 | (23) |
| 5 | 162835 | NZ_CP043667.1 | 4,851,336 | Israel | 2015 | Animal | 31,699 | (23) |
| 6 | CVM 30177 | NZ_CP051346.1 | 4,763,422 | USA | 2003 | Chicken | 4,025 | None |
| 7 | YZ20MCS16 | NZ_CP077680.1 | 4,980,943 | China | 2020 | Market chicken | **No *yidY*** | (16) |
| 8 | ZTA19/00847 | NZ_CP089788.1 | 4838525 | Spain | 2017 | Broiler | **4,744,446** | (22) |
| 9 | ZTA19/00831 | NZ_CP089789.1 | 4837844 | Spain | 2016 | Turkey | 37,133 | (22) |
| 10 | ZTA19/00830 | NZ_CP089790.1 | 4836593 | Spain | 2016 | Turkey | 37,135 | (22) |
| 11 | ZTA19/00820 | NZ_CP089791.1 | 4822417 | Spain | 2015 | Broiler | **No *yidY*** | (22) |
| 12 | ZTA19/00816 | NZ_CP089792.1 | 4834370 | Spain | 2015 | Turkey | 37,135 | (22) |
| 13 | ZTA19/00814 | NZ_CP089794.1 | 4837091 | Spain | 2015 | Turkey | 37,138 | (22) |
| 14 | ZTA19/00813 | NZ_CP089795.1 | 4884180 | Spain | 2015 | Broiler | 40,831 | (22) |
| 15 | ZTA19/00790 | NZ_CP089797.1 | 4823610 | Spain | 2013 | Broiler | **No *yidY*** | (22) |
| 16 | ZTA19/00789 | NZ_CP089798.1 | 4837742 | Spain | 2013 | Turkey | 37,139 | (22) |
| 17 | ZTA19/00785 | NZ_CP089799.1 | 4837105 | Spain | 2012 | Laying hen | 37,139 | (22) |
| 18 | N18-2092 | NZ_CP091998.1 | 4838258 | Switzerland | 2018 | Human urine | **188,913** | (24) |
| 19 | N20-2289 | NZ_CP091997.1 | 4828508 | Switzerland | 2020 | Human urine | **No *yidY*** | (24) |
| 20 | N16-1393 | NZ_CP091999.1 | 4800346 | Switzerland | 2016 | Human feces | **No *yidY*** | (24) |
| 21 | N14-1660 | NZ_CP092004.1 | 4870154 | Switzerland | 2014 | Human feces | 31,710 | (24) |
| 22 | N12-0931 | NZ_CP092009.1 | 4859922 | Switzerland | 2012 | Human inguinal swab | 35,141 | (24) |
| 23 | N12-0259 | NZ_CP092012.1 | 4867746 | Switzerland | 2012 | Human feces | 31,710 | (24) |
| 24 | KCID6 | NZ_CP101647.1 | 4809621 | Indonesia, Bogor | 2022 | Chicken carcass | 44,985 | None |
| 25 | BCID6 | NZ_CP101648.1 | 4809636 | Indonesia, Bogor | 2022 | Chicken carcass | 44,984 | None |
| 26 | AH19MCS1 | NZ_CP102719.1 | 4948090 | China, Anhui | 2019 | Chicken meat product | 34,368 | (25) |
| 27 | AH19MCS8 | NZ_CP102739.1 | 4882250 | China, Anhui | 2019 | Chicken meat product | 32,606 | (25) |
| 28 | AH19MCS11 | NZ_CP102756.1 | 4905153 | China, Anhui | 2019 | Chicken meat product | 32,674 | (25) |
| 29 | Sal-FJ2064 | NZ_CP104049.1 | 4888433 | China, Fujian | 2020 | Human | 34,367 | None |
| 30 | SA20030505 | NZ_CP022500.1 | 4782363 | Canada, Ontario | 2002 | Gallus gallus, cecal content | 4,025 | None |

**Table S5.** Pairwise SNP distance among the 30 isolates of *S.* Kentucky. The samples NZ_CP022500.1 and NZ_CP051346.1 belonged to ST152. The others belonged to ST198.

|  | NZ_CP092009.1 | NZ_CP089799.1 | NZ_CP089792.1 | NZ_CP089790.1 | NZ_CP089794.1 | NZ_CP089789.1 | NZ_CP089788.1 | NZ_CP089798.1 | NZ_CP089795.1 | NZ_CP089797.1 | NZ_CP089791.1 | NZ_CP102756.1 | NZ_CP102739.1 | NZ_CP104049.1 | NZ_CP102719.1 | SSSE-03 | SSSE-01 | NZ_CP092012.1 | NZ_CP092004.1 | NZ_CP043664.1 | NZ_CP043667.1 | NZ_CP101648.1 | NZ_CP101647.1 | NZ_CP091999.1 | NZ_CP077680.1 | NZ_CP091998.1 | NZ_CP091997.1 | NZ_CP026327.1 | NZ_CP022500.1 | NZ_CP051346.1 |
| --- | --- | --- | --- | --- | --- | --- | --- | --- | --- | --- | --- | --- | --- | --- | --- | --- | --- | --- | --- | --- | --- | --- | --- | --- | --- | --- | --- | --- | --- | --- |
| NZ_CP092009.1 | 0 |  |  |  |  |  |  |  |  |  |  |  |  |  |  |  |  |  |  |  |  |  |  |  |  |  |  |  |  |  |
| NZ_CP089799.1 | 27 | 0 |  |  |  |  |  |  |  |  |  |  |  |  |  |  |  |  |  |  |  |  |  |  |  |  |  |  |  |  |
| NZ_CP089792.1 | 73 | 56 | 0 |  |  |  |  |  |  |  |  |  |  |  |  |  |  |  |  |  |  |  |  |  |  |  |  |  |  |  |
| NZ_CP089790.1 | 70 | 53 | 23 | 0 |  |  |  |  |  |  |  |  |  |  |  |  |  |  |  |  |  |  |  |  |  |  |  |  |  |  |
| NZ_CP089794.1 | 61 | 48 | 24 | 21 | 0 |  |  |  |  |  |  |  |  |  |  |  |  |  |  |  |  |  |  |  |  |  |  |  |  |  |
| NZ_CP089789.1 | 64 | 51 | 27 | 24 | 9 | 0 |  |  |  |  |  |  |  |  |  |  |  |  |  |  |  |  |  |  |  |  |  |  |  |  |
| NZ_CP089788.1 | 58 | 45 | 21 | 18 | 3 | 6 | 0 |  |  |  |  |  |  |  |  |  |  |  |  |  |  |  |  |  |  |  |  |  |  |  |
| NZ_CP089798.1 | 57 | 44 | 20 | 17 | 4 | 7 | 1 | 0 |  |  |  |  |  |  |  |  |  |  |  |  |  |  |  |  |  |  |  |  |  |  |
| NZ_CP089795.1 | 107 | 94 | 72 | 69 | 60 | 63 | 57 | 56 | 0 |  |  |  |  |  |  |  |  |  |  |  |  |  |  |  |  |  |  |  |  |  |
| NZ_CP089797.1 | 64 | 53 | 29 | 26 | 17 | 20 | 14 | 13 | 63 | 0 |  |  |  |  |  |  |  |  |  |  |  |  |  |  |  |  |  |  |  |  |
| NZ_CP089791.1 | 70 | 59 | 35 | 32 | 23 | 26 | 20 | 19 | 69 | 8 | 0 |  |  |  |  |  |  |  |  |  |  |  |  |  |  |  |  |  |  |  |
| NZ_CP102756.1 | 93 | 94 | 70 | 67 | 58 | 61 | 55 | 54 | 104 | 61 | 67 | 0 |  |  |  |  |  |  |  |  |  |  |  |  |  |  |  |  |  |  |
| NZ_CP102739.1 | 88 | 89 | 65 | 62 | 53 | 56 | 50 | 49 | 99 | 56 | 62 | 17 | 0 |  |  |  |  |  |  |  |  |  |  |  |  |  |  |  |  |  |
| NZ_CP104049.1 | 84 | 85 | 61 | 58 | 49 | 52 | 46 | 45 | 95 | 52 | 58 | 29 | 24 | 0 |  |  |  |  |  |  |  |  |  |  |  |  |  |  |  |  |
| NZ_CP102719.1 | 83 | 84 | 60 | 57 | 48 | 51 | 45 | 44 | 94 | 51 | 57 | 28 | 23 | 15 | 0 |  |  |  |  |  |  |  |  |  |  |  |  |  |  |  |
| SSSE-03 | 63 | 64 | 40 | 37 | 28 | 31 | 25 | 24 | 74 | 31 | 37 | 48 | 43 | 39 | 38 | 0 |  |  |  |  |  |  |  |  |  |  |  |  |  |  |
| SSSE-01 | 79 | 80 | 56 | 53 | 44 | 47 | 41 | 40 | 90 | 47 | 53 | 64 | 59 | 55 | 54 | 16 | 0 |  |  |  |  |  |  |  |  |  |  |  |  |  |
| NZ_CP092012.1 | 61 | 68 | 44 | 41 | 32 | 35 | 29 | 28 | 78 | 35 | 41 | 64 | 59 | 55 | 54 | 34 | 50 | 0 |  |  |  |  |  |  |  |  |  |  |  |  |
| NZ_CP092004.1 | 63 | 70 | 46 | 43 | 34 | 37 | 31 | 30 | 80 | 37 | 43 | 66 | 61 | 57 | 56 | 36 | 52 | 6 | 0 |  |  |  |  |  |  |  |  |  |  |  |
| NZ_CP043664.1 | 66 | 73 | 49 | 46 | 37 | 40 | 34 | 33 | 83 | 40 | 46 | 69 | 64 | 60 | 59 | 39 | 55 | 21 | 23 | 0 |  |  |  |  |  |  |  |  |  |  |
| NZ_CP043667.1 | 61 | 68 | 44 | 41 | 32 | 35 | 29 | 28 | 78 | 35 | 41 | 64 | 59 | 55 | 54 | 34 | 50 | 16 | 18 | 21 | 0 |  |  |  |  |  |  |  |  |  |
| NZ_CP101648.1 | 84 | 91 | 67 | 64 | 55 | 58 | 52 | 51 | 101 | 58 | 64 | 87 | 82 | 78 | 77 | 57 | 73 | 53 | 55 | 58 | 53 | 0 |  |  |  |  |  |  |  |  |
| NZ_CP101647.1 | 85 | 92 | 68 | 65 | 56 | 59 | 53 | 52 | 102 | 59 | 65 | 88 | 83 | 79 | 78 | 58 | 74 | 54 | 56 | 59 | 54 | 7 | 0 |  |  |  |  |  |  |  |
| NZ_CP091999.1 | 83 | 90 | 66 | 63 | 54 | 57 | 51 | 50 | 100 | 57 | 63 | 86 | 81 | 77 | 76 | 56 | 72 | 51 | 53 | 56 | 51 | 69 | 70 | 0 |  |  |  |  |  |  |
| NZ_CP077680.1 | 88 | 95 | 71 | 68 | 59 | 62 | 56 | 55 | 105 | 62 | 68 | 91 | 86 | 82 | 81 | 61 | 77 | 56 | 58 | 61 | 56 | 74 | 75 | 31 | 0 |  |  |  |  |  |
| NZ_CP091998.1 | 69 | 76 | 52 | 49 | 40 | 43 | 37 | 36 | 86 | 43 | 49 | 72 | 67 | 63 | 62 | 42 | 58 | 37 | 39 | 42 | 37 | 55 | 56 | 50 | 55 | 0 |  |  |  |  |
| NZ_CP091997.1 | 74 | 81 | 57 | 54 | 45 | 48 | 42 | 41 | 91 | 48 | 54 | 77 | 72 | 68 | 67 | 47 | 63 | 42 | 44 | 47 | 42 | 60 | 61 | 55 | 60 | 9 | 0 |  |  |  |
| NZ_CP026327.1 | 95 | 102 | 78 | 75 | 66 | 69 | 63 | 62 | 112 | 69 | 75 | 98 | 93 | 89 | 88 | 68 | 84 | 63 | 65 | 68 | 63 | 81 | 82 | 76 | 81 | 36 | 41 | 0 |  |  |
| NZ_CP022500.1 | 43679 | 43684 | 43680 | 43677 | 43668 | 43671 | 43665 | 43664 | 43712 | 43671 | 43677 | 43702 | 43695 | 43693 | 43692 | 43672 | 43685 | 43667 | 43669 | 43672 | 43667 | 43685 | 43686 | 43682 | 43687 | 43668 | 43673 | 43652 | 0 |  |
| NZ_CP051346.1 | 43661 | 43666 | 43666 | 43663 | 43654 | 43657 | 43651 | 43650 | 43698 | 43657 | 43663 | 43688 | 43681 | 43679 | 43678 | 43658 | 43671 | 43653 | 43655 | 43658 | 43653 | 43671 | 43672 | 43668 | 43673 | 43654 | 43659 | 43638 | 215 | 0 |

**Table S6.** The presence of specific antimicrobial resistance genes of 28 *S.* Kentucky ST198 isolates. The number 1 and 0 indicate the presence and absence of each gene respectively. The positions of the mutations in GyrA and ParC are indicated.

|  | **Aminoglycoside** | | | | | | | | | | | | | | | **Sulphona-mide** | | **Tetracycline** | **Beta-lactam** | | | **Phenicol** | | | **Fosfomycin** | **Quinolone** | | **Quinolone**  **(target mutation)** | | | | **Macrolide/**  **Lincosamide** | | | | **Trimetho-prim** | | **Rifampicin** | **Colistin** |
| --- | --- | --- | --- | --- | --- | --- | --- | --- | --- | --- | --- | --- | --- | --- | --- | --- | --- | --- | --- | --- | --- | --- | --- | --- | --- | --- | --- | --- | --- | --- | --- | --- | --- | --- | --- | --- | --- | --- | --- |
| **Strain** | ***aac(6')-Iaa*** | ***aadA7*** | ***aadA17*** | ***aadA1*** | ***aadA2*** | ***aac(3)-Iid*** | ***aac(3)-IV*** | ***aac(3)-Id*** | ***aph(6)-Id*** | ***aac(6')-Ib3*** | ***aph(3')-Ia*** | ***aph(4)-Ia*** | ***aph(3'')-Ib*** | ***armA*** | ***rmtB*** | ***sul1*** | ***sul3*** | ***tet(A)*** | ***bla_TEM-1B_*** | ***bla_CTX-M-55_*** | ***bla_CTX-M-14b_*** | ***cmlA1*** | ***floR*** | ***catA1*** | ***fosA3*** | ***qnrS1*** | ***aac(6')-Ib-cr*** | **GyrA** | | **ParC** | | ***lnu(F)*** | ***mph(A)*** | ***mph(E)*** | ***msr(E)*** | ***dfrA14*** | ***drfA12*** | ***ARR-2*** | ***mcr-1.1*** |
| **NZ_CP092009.1** | 1 | 0 | 0 | 0 | 0 | 0 | 0 | 0 | 0 | 1 | 0 | 0 | 0 | 1 | 0 | 1 | 0 | 1 | 1 | 0 | 0 | 0 | 0 | 0 | 0 | 0 | 1 | S83F | D87N | T57S | S80I | 0 | 0 | 1 | 1 | 0 | 0 | 0 | 0 |
| **NZ_CP089799.1** | 1 | 1 | 0 | 0 | 0 | 0 | 0 | 1 | 0 | 0 | 0 | 0 | 0 | 0 | 0 | 1 | 0 | 0 | 0 | 0 | 0 | 0 | 0 | 0 | 0 | 0 | 0 | S83F | D87N | T57S | S80I | 0 | 0 | 0 | 0 | 0 | 0 | 0 | 0 |
| **NZ_CP089792.1** | 1 | 1 | 0 | 0 | 0 | 0 | 0 | 1 | 0 | 0 | 0 | 0 | 0 | 0 | 0 | 1 | 0 | 0 | 0 | 0 | 0 | 0 | 0 | 0 | 0 | 0 | 0 | S83F | D87N | T57S | S80I | 0 | 0 | 0 | 0 | 0 | 0 | 0 | 0 |
| **NZ_CP089790.1** | 1 | 1 | 0 | 0 | 0 | 0 | 0 | 1 | 0 | 0 | 0 | 0 | 0 | 0 | 0 | 1 | 0 | 0 | 0 | 0 | 0 | 0 | 0 | 0 | 0 | 0 | 0 | S83F | D87N | T57S | S80I | 0 | 0 | 0 | 0 | 0 | 0 | 0 | 0 |
| **NZ_CP089794.1** | 1 | 1 | 0 | 0 | 0 | 0 | 0 | 1 | 0 | 0 | 0 | 0 | 0 | 0 | 0 | 1 | 0 | 0 | 0 | 0 | 0 | 0 | 0 | 0 | 0 | 0 | 0 | S83F | D87N | T57S | S80I | 0 | 0 | 0 | 0 | 0 | 0 | 0 | 0 |
| **NZ_CP089789.1** | 1 | 1 | 0 | 0 | 0 | 0 | 0 | 1 | 0 | 0 | 0 | 0 | 0 | 0 | 0 | 1 | 0 | 0 | 0 | 0 | 0 | 0 | 0 | 0 | 0 | 0 | 0 | S83F | D87N | T57S | S80I | 0 | 0 | 0 | 0 | 0 | 0 | 0 | 0 |
| **NZ_CP089788.1** | 1 | 1 | 0 | 0 | 0 | 0 | 0 | 1 | 0 | 0 | 0 | 0 | 0 | 0 | 0 | 1 | 0 | 0 | 0 | 0 | 0 | 0 | 0 | 0 | 0 | 0 | 0 | S83F | D87N | T57S | S80I | 0 | 0 | 0 | 0 | 0 | 0 | 0 | 0 |
| **NZ_CP089798.1** | 1 | 1 | 0 | 0 | 0 | 0 | 0 | 1 | 0 | 0 | 0 | 0 | 0 | 0 | 0 | 1 | 0 | 0 | 0 | 0 | 0 | 0 | 0 | 0 | 0 | 0 | 0 | S83F | D87N | T57S | S80I | 0 | 0 | 0 | 0 | 0 | 0 | 0 | 0 |
| **NZ_CP089795.1** | 1 | 1 | 0 | 0 | 0 | 0 | 0 | 1 | 0 | 0 | 0 | 0 | 0 | 0 | 0 | 1 | 0 | 0 | 1 | 0 | 0 | 0 | 0 | 0 | 0 | 0 | 0 | S83F | D87N | T57S | S80I | 0 | 0 | 0 | 0 | 0 | 0 | 0 | 0 |
| **NZ_CP089797.1** | 1 | 0 | 0 | 0 | 0 | 0 | 0 | 0 | 0 | 0 | 0 | 0 | 0 | 0 | 0 | 0 | 0 | 0 | 0 | 0 | 0 | 0 | 0 | 0 | 0 | 0 | 0 | S83F | D87N | T57S | S80I | 0 | 0 | 0 | 0 | 0 | 0 | 0 | 0 |
| [**NZ_CP089791.1**](https://www.ncbi.nlm.nih.gov/nuccore/NZ_CP089791.1/) | 1 | 0 | 0 | 0 | 0 | 0 | 0 | 0 | 0 | 0 | 0 | 0 | 0 | 0 | 0 | 0 | 0 | 0 | 0 | 0 | 0 | 0 | 0 | 0 | 0 | 0 | 0 | S83F | D87N | T57S | S80I | 0 | 0 | 0 | 0 | 0 | 0 | 0 | 0 |
| [**NZ_CP102756.1**](https://www.ncbi.nlm.nih.gov/nuccore/NZ_CP102756.1/) | 1 | 0 | 1 | 0 | 0 | 1 | 0 | 0 | 0 | 0 | 1 | 0 | 0 | 0 | 1 | 1 | 0 | 1 | 1 | 1 | 0 | 0 | 1 | 0 | 0 | 0 | 0 | S83F | D87N | T57S | S80I | 1 | 1 | 0 | 0 | 1 | 0 | 1 | 0 |
| [**NZ_CP102739.1**](https://www.ncbi.nlm.nih.gov/nuccore/NZ_CP102739.1/) | 1 | 1 | 1 | 0 | 0 | 1 | 0 | 1 | 0 | 0 | 1 | 0 | 0 | 0 | 1 | 1 | 0 | 1 | 1 | 1 | 0 | 0 | 1 | 0 | 1 | 0 | 0 | S83F | D87N | T57S | S80I | 1 | 0 | 0 | 0 | 1 | 0 | 1 | 0 |
| **NZ_CP104049.1** | 1 | 1 | 1 | 0 | 0 | 1 | 0 | 1 | 0 | 0 | 1 | 0 | 0 | 0 | 1 | 1 | 0 | 1 | 1 | 1 | 0 | 0 | 1 | 0 | 1 | 1 | 0 | S83F | D87N | T57S | S80I | 1 | 1 | 0 | 0 | 1 | 0 | 1 | 0 |
| [**NZ_CP102719.1**](https://www.ncbi.nlm.nih.gov/nuccore/NZ_CP102719.1/) | 1 | 1 | 1 | 0 | 0 | 1 | 1 | 1 | 0 | 0 | 1 | 1 | 0 | 0 | 1 | 1 | 0 | 1 | 1 | 1 | 0 | 0 | 1 | 0 | 1 | 1 | 0 | S83F | D87N | T57S | S80I | 1 | 1 | 0 | 0 | 0 | 0 | 0 | 0 |
| **SSSE03** | 1 | 1 | 1 | 0 | 0 | 1 | 0 | 1 | 0 | 0 | 0 | 0 | 0 | 0 | 0 | 1 | 0 | 1 | 1 | 0 | 0 | 0 | 0 | 0 | 0 | 0 | 0 | S83F | D87N | T57S | S80I | 1 | 0 | 0 | 0 | 0 | 0 | 0 | 0 |
| **SSSE01** | 1 | 1 | 1 | 0 | 0 | 1 | 0 | 1 | 0 | 0 | 0 | 0 | 0 | 0 | 0 | 1 | 0 | 1 | 1 | 0 | 0 | 0 | 0 | 0 | 0 | 0 | 0 | S83F | D87N | T57S | S80I | 1 | 0 | 0 | 0 | 0 | 0 | 0 | 0 |
| **NZ_CP092012.1** | 1 | 1 | 0 | 0 | 0 | 0 | 0 | 1 | 0 | 0 | 0 | 0 | 0 | 0 | 0 | 1 | 0 | 1 | 1 | 0 | 0 | 0 | 0 | 0 | 0 | 0 | 0 | S83F | D87Y | T57S | S80I | 0 | 0 | 0 | 0 | 0 | 0 | 0 | 0 |
| [**NZ_CP092004.1**](https://www.ncbi.nlm.nih.gov/nuccore/NZ_CP092004.1/) | 1 | 1 | 0 | 0 | 0 | 0 | 0 | 1 | 0 | 0 | 0 | 0 | 0 | 0 | 0 | 1 | 0 | 1 | 1 | 0 | 0 | 0 | 0 | 0 | 0 | 0 | 0 | S83F | D87Y | T57S | S80I | 0 | 0 | 0 | 0 | 0 | 0 | 0 | 0 |
| **NZ_CP043664.1** | 1 | 1 | 0 | 0 | 0 | 0 | 0 | 1 | 0 | 0 | 0 | 0 | 0 | 0 | 0 | 1 | 0 | 1 | 1 | 0 | 0 | 0 | 0 | 0 | 0 | 0 | 0 | S83F | D87Y | T57S | S80I | 0 | 0 | 0 | 0 | 0 | 0 | 0 | 0 |
| **NZ_CP043667.1** | 1 | 0 | 0 | 0 | 0 | 0 | 0 | 0 | 0 | 0 | 0 | 0 | 0 | 0 | 0 | 0 | 0 | 0 | 1 | 0 | 0 | 0 | 0 | 0 | 0 | 0 | 0 | S83F | D87Y | T57S | S80I | 0 | 0 | 0 | 0 | 0 | 0 | 0 | 0 |
| [**NZ_CP101648.1**](https://www.ncbi.nlm.nih.gov/nuccore/NZ_CP101648.1/) | 1 | 1 | 0 | 0 | 0 | 0 | 0 | 1 | 0 | 0 | 0 | 0 | 0 | 0 | 0 | 1 | 0 | 1 | 0 | 0 | 0 | 0 | 0 | 0 | 0 | 0 | 0 | S83F | D87N | T57S | S80I | 0 | 0 | 0 | 0 | 0 | 0 | 0 | 0 |
| [**NZ_CP101647.1**](https://www.ncbi.nlm.nih.gov/nuccore/NZ_CP101647.1/) | 1 | 1 | 0 | 0 | 0 | 0 | 0 | 1 | 0 | 0 | 0 | 0 | 0 | 0 | 0 | 1 | 0 | 1 | 0 | 0 | 0 | 0 | 0 | 0 | 0 | 0 | 0 | S83F | D87N | T57S | S80I | 0 | 0 | 0 | 0 | 0 | 0 | 0 | 0 |
| [**NZ_CP091999.1**](https://www.ncbi.nlm.nih.gov/nuccore/NZ_CP091999.1/) | 1 | 0 | 0 | 0 | 0 | 0 | 0 | 0 | 0 | 0 | 1 | 0 | 0 | 0 | 0 | 0 | 0 | 0 | 0 | 0 | 1 | 0 | 0 | 0 | 0 | 0 | 0 | S83F | D87G | T57S | S80I | 0 | 0 | 0 | 0 | 0 | 0 | 0 | 0 |
| **NZ_CP077680.1** | 1 | 1 | 0 | 0 | 0 | 0 | 0 | 1 | 1 | 0 | 1 | 0 | 1 | 0 | 0 | 1 | 0 | 1 | 0 | 0 | 1 | 0 | 0 | 0 | 0 | 0 | 0 | S83F | D87G | T57S | S80I | 0 | 0 | 0 | 0 | 0 | 0 | 0 | 1 |
| [**NZ_CP091998.1**](https://www.ncbi.nlm.nih.gov/nuccore/NZ_CP091998.1/) | 1 | 0 | 0 | 0 | 1 | 0 | 0 | 0 | 0 | 0 | 0 | 0 | 0 | 0 | 0 | 1 | 0 | 0 | 1 | 0 | 0 | 0 | 0 | 1 | 0 | 0 | 0 | S83F | D87G | T57S | S80I | 0 | 1 | 0 | 0 | 0 | 1 | 0 | 0 |
| **NZ_CP091997.1** | 1 | 0 | 0 | 0 | 1 | 0 | 0 | 0 | 0 | 0 | 0 | 0 | 0 | 0 | 0 | 1 | 0 | 0 | 0 | 0 | 0 | 0 | 0 | 0 | 0 | 0 | 0 | S83F | D87G | T57S | S80I | 0 | 1 | 0 | 0 | 0 | 1 | 0 | 0 |
| **NZ_CP026327.1** | 1 | 0 | 0 | 1 | 1 | 0 | 0 | 0 | 0 | 0 | 1 | 0 | 0 | 0 | 0 | 1 | 1 | 1 | 1 | 0 | 0 | 1 | 0 | 0 | 0 | 0 | 0 | S83F | D87G | T57S | S80I | 0 | 1 | 0 | 0 | 0 | 1 | 0 | 0 |

**Supplementary Figure**


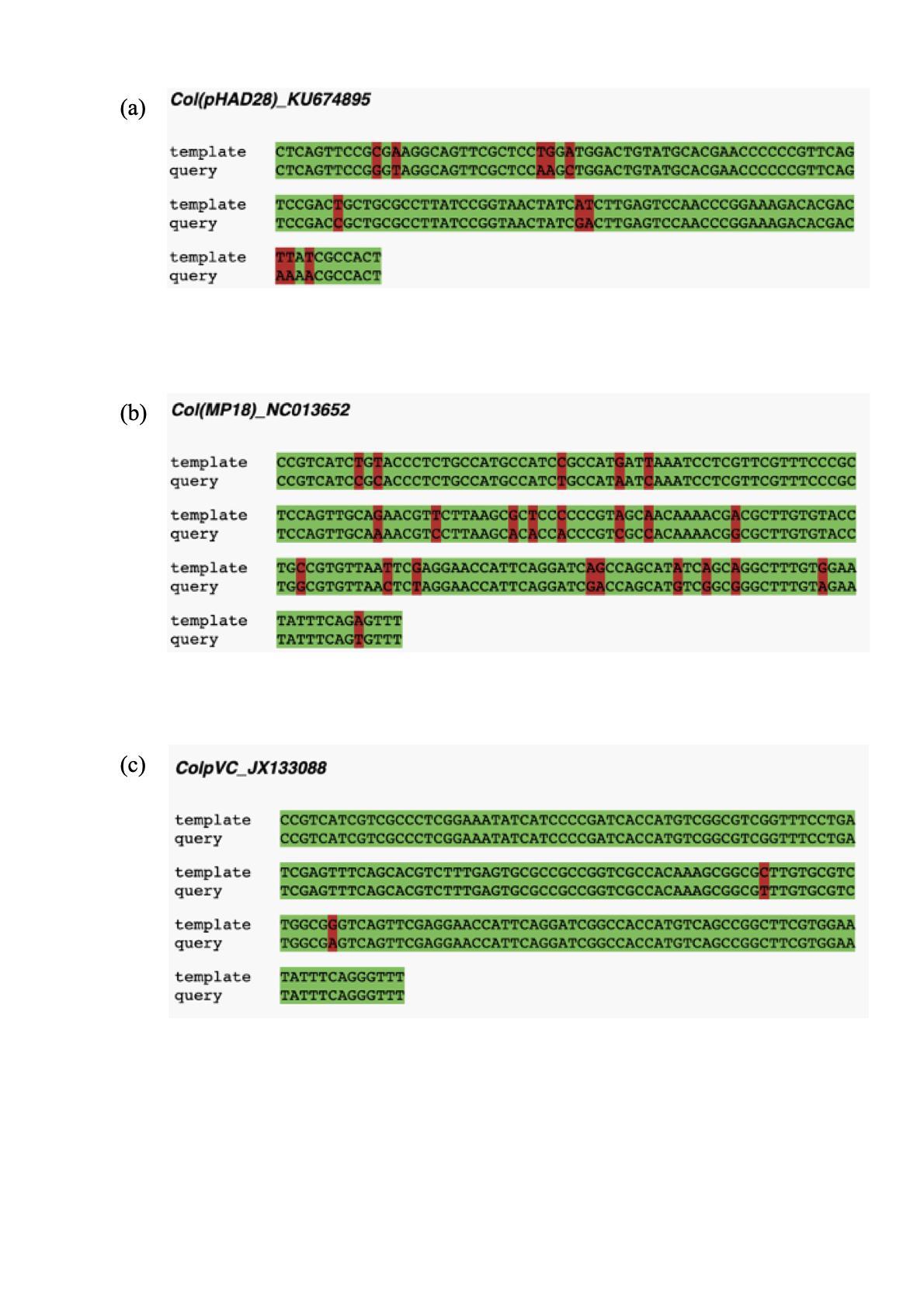


**Fig S1.** Alignment of the replicons of the three plasmids in *S.* Kentucky ST198 SSSE-01 compared to the most similar ones identified by Plasmidfinder: (a) pSSSE-01a, (b) pSSSE-01b and (c) pSSSE-01c. The names and accession numbers of the most similar reference sequence files are indicated. The template refers to the reference sequences. The queries are the sequences of pSSSE-01, which are identical to the ones in pSSSE-03. The mismatched bases are highlighted in red.
